# Authentication of *Allium ulleungense*, *A*. *microdictyon* and *A*. *ochotense* based on super-barcoding of plastid genome and 45S nrDNA

**DOI:** 10.1371/journal.pone.0294457

**Published:** 2023-11-20

**Authors:** Minyoung Lee, Hyo Young Lee, Jong-Soo Kang, Hyeji Lee, Ki-Jin Park, Jee Young Park, Tae-Jin Yang

**Affiliations:** 1 Department of Agriculture, Plant Genomics & Breeding Institute, Research Institute of Agriculture and Life Science, Forestry and Bioresources, College of Agriculture & Life Sciences, Seoul National University, Seoul, Republic of Korea; 2 Gangwondo State Agricultural Research & Extension Services, Wild Vegetable Research Institute, Pyeongchang-gun, Gangwon State, Republic of Korea; University of Nebraska-Lincoln, UNITED STATES

## Abstract

*Allium ulleungense* (*AU*) and *A*. *microdictyon* (*AM*) are valuable medicinal and edible vegetables, referred to as mountain garlic in Korea. The identification of *AU*, *AM* and a neighboring species *A*. *ochotense* (*AO*) is difficult because of their morphological similarities. We collected samples from three species and 46 cultivated collections to understand the genetic diversity of these valuable *Allium* species. Among them, we sequenced six collections, including three species and three cultivating collections to obtain data from the plastid genome (plastome) and nuclear 45S ribosomal DNA (nrDNA) for super-barcoding. The *AM* and *AO* showed around 60 single nucleotide polymorphisms (SNPs) and 39 Insertion/Deletion (InDels) in the plastome but no variations in the nrDNA sequences. Conversely, the *AU* and *AM* showed more than 170 SNPs and 80 InDels in the plastomes, and 20 SNPs and 1 InDel were found in the 45S nrDNA sequences. Among the three cultivating collections, one TB collection was determined to be the *AU* type in both plastome and nrDNA sequences. However, the other two collections, JB and SA, showed the *AM* type plastome but were heterozygous in the 45S nrDNA sequences, indicating both *AU* and *AM* types (putative *AM* x *AU* hybrid). Ten molecular markers were developed based on sequence variations to identify these three species and assess their genetic diversity. A total of 49 collections were genotyped using the ten developed markers and classified into five groups: 14 *AU*, 22 *AM*, 1 *AO*, 3 putative *AM* x *AU* hybrids, and 9 putative *AU* x *AM* hybrid collections. Super-barcoding with plastomes and nrDNAs revealed the genetic diversity of the three *Allium* species and putative hybrids between species. The newly developed markers will facilitate species and hybrid identification, thereby benefiting marker-assisted molecular breeding of *Allium* species.

## Introduction

*Allium ulleungense* (*AU*), *A*. *ochotense* (*AO*) and *A*. *microdictyon* (*AM*) that belong to subgenus *Anguinum*, are commonly known as ‘Siberian garlic’ or ‘Alpine leek’. They are widely used as a vegetable, called mountain garlic, due to their strong garlic-like odor and taste. These *Allium* species are known to have medicinal functions such as anti-cancer, anti-inflammatory and anti-oxidant [1–4]. Due to their morphological similarity, it is not easy to distinguish these three *Allium* species in the farm fields and agricultural market. However, the three *Allium* species can be distinguished by ploidy level, distribution, taste and growth. The *AO* is tetraploid (2n = 4x = 32) and is distributed in Japan, Northeast China, Southeast Russia and North Korea [5]. On the other hand, *AM* and *AU* are diploid (2n = 2x = 16) [5]. The *AM* is distributed in Siberia, the Southern Ural area, North Mongolia, Kazakhstan and Korea [5,6], and *AU* is endemic to the Ulleung Island of South Korea. The *AU* was recently treated as an independent species in South Korea [7]. The *AU* can be distinguished from *AM* by its broader leaves and larger whitish perianth [7]. In agricultural markets, *AM* is usually preferred by customers because of its flavor, taste and texture, compared with *AU*. However, the productivity of *AM* is lower than that of *AU* in farm fields. Therefore, these three *Allium* species need to be studied for species identification and for the molecular breeding of a new *Allium* cultivar with the better taste of *AM* and excellent productivity of *AU*.

In order to ensure accurate identification and efficient breeding of these species, analyzing molecular data is necessary to assess the genetic diversity of *Allium* species. In general, universal barcoding regions are commonly used for the molecular identification of plants. However, molecular identification using universal barcoding regions often provides insufficient data to identify the species, especially when two or more species are close and the barcoding region is conserved in the group [8]. Thus, super-barcoding, using complete plastid genomes (plastomes) and entire nuclear ribosomal DNA (nrDNA), is considered a powerful tool to identify the closely related species, by providing abundant genetic variation [9–11]. Due to the advances of next-generation sequencing, a large amount of plastome data in land plants has been reported and is available in NCBI GenBank [10], allowing us to assess the genetic diversity of the species from various locations.

In this study, we newly sequenced three *Allium* species (*AU*, *AM*, and *AO*) to obtain plastome and nrDNA data and then investigated variations among the three species to develop molecular markers for identifying the three *Allium* species and assessing the genetic diversity. The developed molecular markers were verified with 46 collections of the three species and demonstrated to be useful in identifying the species and assessing the genetic diversity. We believe that the developed markers in this study will be useful for the molecular breeding of the *Allium* species.

## Materials and methods

### Plant materials and genomic DNA extraction

Plant materials of six *Allium* samples were provided from the Gangwondo Agricultural Research & Extension Services, Wild Vegetable Research Institute, Republic of Korea (Table 1, S1 Fig), and no specific permission was required for collecting the species in this study, according to the national and local legislations. Specimens of the six *Allium* samples used in this study were deposited in the T.B. Lee Herbarium of Seoul National University under the voucher numbers SNUA00058899 to SNUA00058905 (Table 1). Leaf materials were ground with liquid nitrogen using a mortar and pestle, and total genomic DNA was extracted using the GeneAll^Ⓡ^ ExgeneTM Plant SV Mini Kit (GeneAll Biotechnology LTD., South Korea) according to the manufacturer’s instructions. The quality and concentration of the extracted DNA were measured using a NanoDrop ND-1000 (Thermo Fisher Scientific, USA).

**Table 1:** Information of assembled plastomes and 45S nrDNAs of six *Allium* samples used in this study.

No.	Species name		Collection site	Voucher number	Abbreviation	Raw data (Gbp)	Plastome		45s nrDNA	
							coverage (x)	length (bp)	coverage (x)	length (bp)
1	*Allium ulleungense*		Ulleung-island, South Korea	SNUA00058905	*AU*	1.07	49.1	154047	812.71	5878
2	*Allium microdictyon*		Heungjeong, Sounth Korea	SNUA00058899	*AM*	1.98	146.57	153562	781.71	5879
3	*Allium ochotense*		Japan	SNUA00058903	*AO*	1.04	74.31	153125	500.33	5879
4	Farm collections	Taebaek	South Korea	SNUA00058904	Farm-TB	1.14	53.99	154051	705.15	5878
5		Jinbu	South Korea	SNUA00058900	Farm-JB	1.07	54.86	153560	451.61	5878
6		Seorak	South Korea	SNUA00058902	Farm-SA	1.04	64	153561	469.73	5878

### Next-generation sequencing and quality trimming

Paired-end library construction and next-generation sequencing (NGS) were conducted using the Illumina MiSeq platform (Illumina, USA) at Phyzen (www.phyzen.com, South Korea). About 1~2 Gbp of raw data were generated from six *Allium* samples (S1 Table). The obtained raw reads were trimmed based on the quality score, maintaining a minimum quality score ≥20, using the CLC quality trim (version 4.06beta.67189, CLC Inc., Denmark).

### Plastome and 45S nuclear ribosomal DNA assembly and annotation

After quality trimming, plastome and 45S nrDNA sequences of six *Allium* samples were assembled using the *de novo* assembly of low-coverage whole-genome sequencing (dnaLCW) method [12,13], in the CLC genome assembler program (ver. 4.6 beta, CLC Inc., Denmark). Trimmed reads were assembled with parameters of overlapping distance ranging from 150 to 500 bp. Plastome contigs were extracted from the assembled sequences using MUMmer, and the *Allium victorialis* plastome sequence (NC_037240) was used as a reference. Extracted plastome contigs were assembled into a single draft sequence, and were manually curated based on mapping results of the NGS reads. The nrDNA contigs were retrieved and assembled in conjunction with the nrDNA sequence of *Brassica oleracea* (KM538957).

Complete plastome sequences were annotated using the GeSeq program (https://chlorobox.mpimp-golm.mpg.de/geseq.html) [14] and manually corrected using the Artemis program [15]. The circular map of plastomes was drawn using the OGDRAW program (https://chlorobox.mpimp-golm.mpg.de/OGDraw.html) [16]. The 45S nrDNA region, including 18S, 5.8S, 26S and two internal transcribed spacer (ITS) regions, was annotated using a BLAST search. The assembled plastome and 45S nrDNA sequences of six *Allium* samples were deposited in the NCBI GenBank under accession numbers OQ183591 to OQ182596 (plastomes), and OQ183920 to OQ183925 (45S nrDNAs), respectively.

### Sequence comparison and molecular marker development

Plastome and nrDNA sequences were aligned using MAFFT (www.mafft.cbrc.jp) [17] with six *Allium* samples. After the alignment, single-nucleotide polymorphisms (SNPs) and insertions/deletions (InDels) were counted and categorized into inter- and intraspecies polymorphisms. To develop molecular markers, InDel regions among the *Allium* plastomes longer than 10 bp were selected to design the codominant marker, and one SNP region was chosen for designing the dominant marker. The SNPs in the 45S nrDNA regions were selected and used for the high-resolution melting curve (HRM) analysis. Primers for codominant, dominant markers and HRM analysis were designed using Primer-BLAST [18] with default parameters.

PCR amplification was conducted in 25-μL reactions containing 1 unit of Taq DNA polymerase (Inclone Biotech, South Korea), 2.5 μL of 10x reaction buffer, 0.2 mM dNTPs, 20 ng genomic DNA, and 10 pM of each primer. The thermal cycling conditions were as follows: pre-amplification at 95°C for 5 min; 35 cycles of denaturation at 95°C for 20 sec, annealing at 58–63°C for 20 sec, and elongation at 72°C for 20 sec; followed by a final elongation at 72°C for 5 min. PCR products were confirmed by electrophoresis using a 3% (w/v) agarose gel.

The nrDNA-based HRM analysis was performed using a LightCycler 96 Real-time PCR Machine (Roche Applied Science, Indianapolis, IN, United States) under the following conditions: pre-amplification at 95°C for 5 min; 45 cycles of 95°C for 20 sec, 60°C for 20 sec, and 72°C for 20 sec; 1 cycle of 95°C for 1 min, 40°C for 1 min, 60°C for 5 sec, 95°C for 5 sec; followed by a cycle of 40°C for 30 sec. The reaction was conducted in 20-μL reactions containing 1 unit of Taq DNA polymerase (Inclone Biotech, South Korea), 2 μL of 10x reaction buffer, 0.2 mM dNTPs, 20 ng genomic DNA, 10 pM of each primer, and 2.5μM SYTO 9 (Thermo Fisher Scientific, USA).

### Phylogenetic analysis

Plastome sequences of six *Allium* samples and 19 previously reported *Allium* plastomes that were downloaded from NCBI GenBank were aligned using MAFFT [17]. For the nrDNA phylogeny, the ITS1-5.8S-ITS2 region of 45S nrDNA of six *Allium* samples and those from 64 previously reported *Allium* individuals downloaded from NCBI were aligned using MAFFT. Phylogenetic analyses based on whole plastome sequences (excluding one copy of IR region) and 45S nrDNA (18S, 5.8S, 26S and two ITS regions) sequences were conducted using RAxML [19] with 1000 bootstrap replicates and a substitution model of GTRGAMMA, respectively.

## Results

### Plastome and nrDNA assemblies

**Fig 1:**
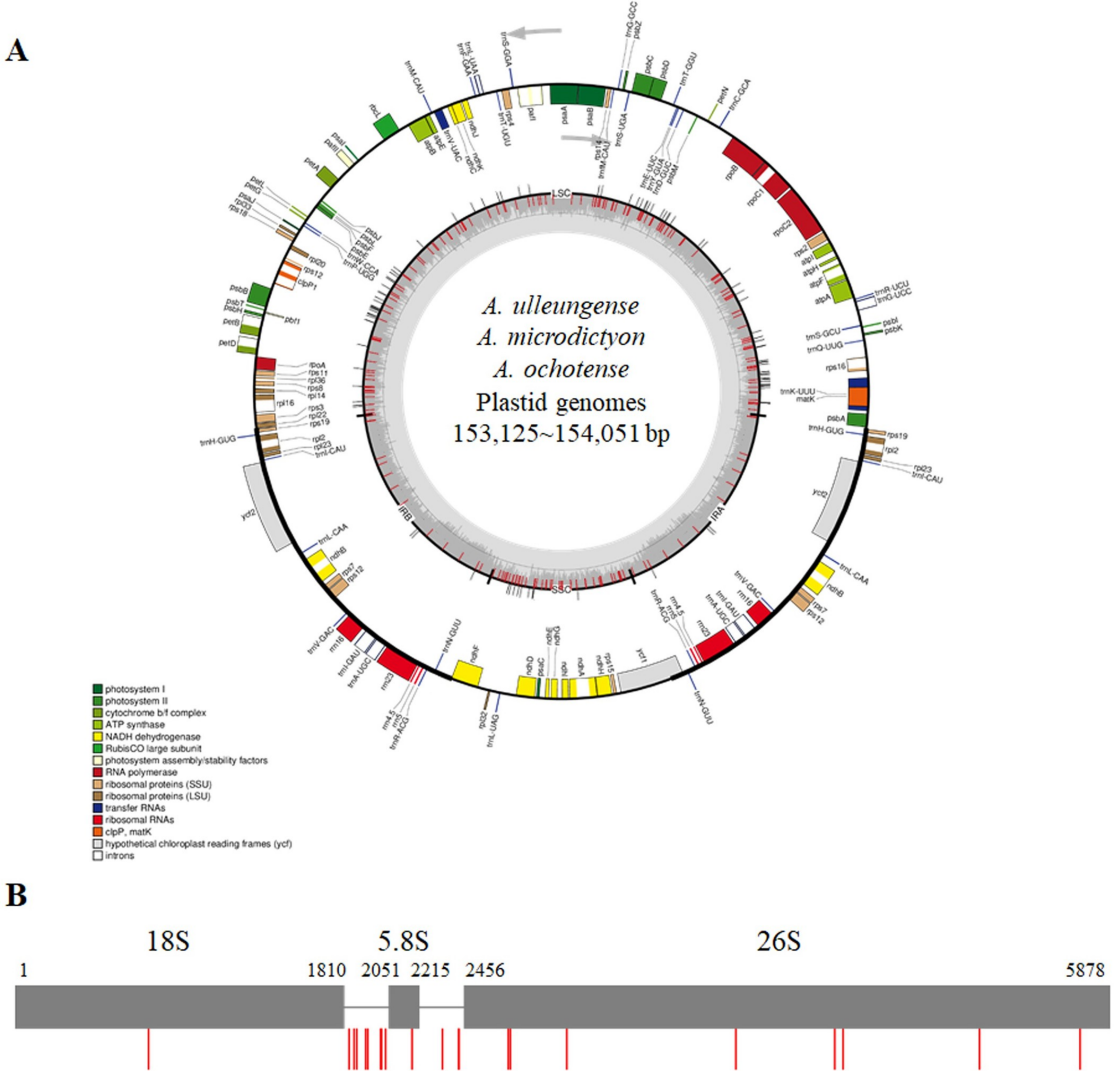
Assembly of six *Allium* plastomes and 45S nrDNA sequences and variation analysis. (A) Circular map of six *Allium* plastomes. The species and total length are written in the center of the circular map. Annotated genes are marked on the outer circle and functions of these genes are categorized based on the legend placed in the bottom left. Red bars of the inner circle indicate SNP positions and black bars of the inner circle indicate InDel positions. (B) Map and variable sites within the 45S nrDNA sequence of six *Allium* samples. Gray boxes indicate transcription unit and red bars indicate variable sites among six *Allium* samples.

Through the Illumina Miseq platform, approximately 1 gbp of high quality reads were used for simultaneous *de novo* assembly of plastomes and nrDNAs (S1 Table). Complete plastomes and 45S nrDNAs of six *Allium* samples were assembled. Total length of the assembled plastomes ranged from 153,125 to 154,051 bp (Table 1). The plastomes of *AU* and Farm-TB were relatively larger than those of *AM*, *AO*, Farm-JB, and Farm-SA. Plastomes consisted of 82,183 to 83,147 bp of large single copy region (LSC), 26,542 to 26,526 bp of the IR, and 17,852 to 17,858 bp of the SSC (S2 Table). All the six *Allium* plastomes contained 86 protein-coding genes, 38 tRNA genes and eight rRNA genes (S2 Table, Fig 1A). No difference in the number of genes among six *Allium* plastomes was found. Total length of 45S nrDNAs ranged from 5,878 to 5,879 bp with coverage of 451.61 to 812.71x (Table 1, Fig 1B). The assembled 45S nrDNAs of *AU*, Farm-TB, Farm-JB and Farm-SA were identical in length with 5,878 bp, and those of *AM* and *AO* were 5,879 bp. A one bp InDel between *AU* and *AM* was found at the 1,936th nucleotide of the 45S nrDNA sequence.

### Intra- and interspecific variations in the plastome and 45S nrDNA

**Fig 2:**
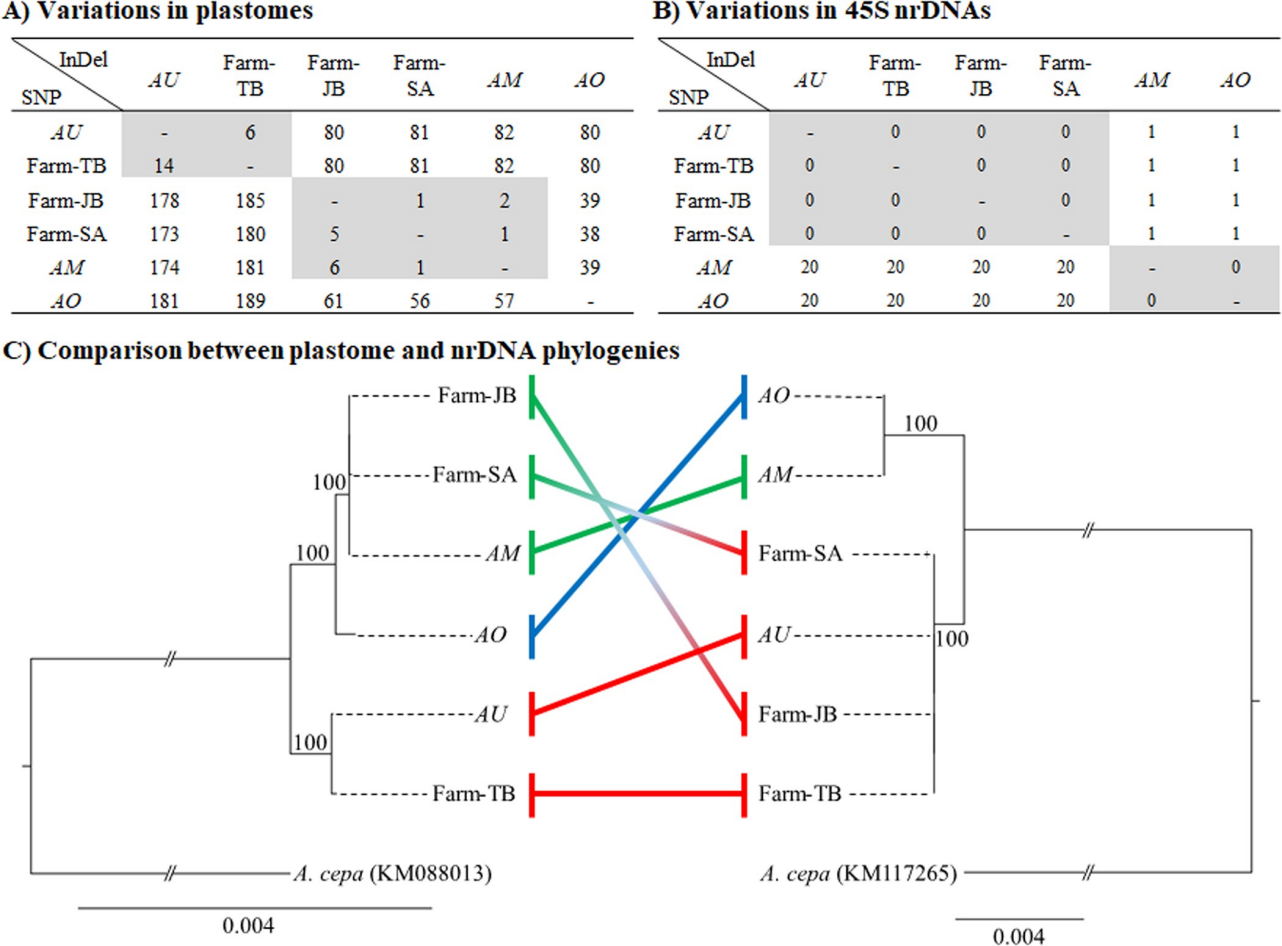
Comparative analysis of six *Allium* plastomes and 45S nrDNA sequences. (A) Variations observed among six *Allium* plastomes. (B) Variations observed among six *Allium* 45S nrDNA sequences. (C) Phylogenetic relationships among six *Allium* samples based on whole plastome sequences (excluding one copy of IR region) and 45S nrDNA sequences. The plastome and 45S nrDNA sequences of *A*. *cepa* were included as the outgroup. The phylogenetic tree on the left is based on plastomes, while the one on the right is based on 45S nrDNA sequences. Bootstrap values greater than 50% are shown near the branches. The colored lines in the middle highlight comparisons between the two trees.

We aligned the plastome and 45S nrDNA sequences of six *Allium* samples, and identified sequence variations among the six *Allium* samples. A total of 14 SNPs and six InDels were identified between the *AU* and Farm-TB plastomes, and less than six SNPs and less than two InDels were detected among the plastomes of *AM*, Farm-JB, and Farm-SA (Fig 2A). The *AO* plastome was distinct from the other five plastomes, containing about 180 SNPs and 80 InDels with the *AU* and Farm-TB plastomes, and about 60 SNPs and 40 InDels with the plastomes of *AM*, Farm-JB, and Farm-SA (Fig 2A). In the plastome-based phylogeny, both *AU* and Farm-TB formed their own clade, which was sister to the clade of *AM*, *AO*, Farm-JB, and Farm-SA (Fig 2C). Within the clade of the four *Allium* samples, the former three formed a subclade, and *AO* was sister to the subclade of three *Allium* samples (Fig 2C).

The nrDNA sequence comparison showed that the nrDNA sequences of *AU* and three farm collections (TB, JB and SA) were identical, and those of *AM* and *AO* were identical (Fig 2B). We found 20 SNPs and one InDel between nrDNA sequences of the two groups (*AU*, Farm-TB, Farm-JB, and Farm-SA vs. *AM* and *AO*) (Fig 2B). Unlike the plastome-based phylogeny, the nrDNA phylogeny showed that *AO* and *AM* formed a subclade, and *AU* and the three farm collections (TB, SA, and JB) formed a subclade (Fig 2C). We found phylogenetic discordance on the phylogenetic positions of farm collections JB and SA in the plastome and nrDNA phylogenies. The two samples formed a clade with *AM* and *AO* in the plastome-based phylogeny, but they formed a clade with *AU* and Farm-TB in the nrDNA phylogeny (Fig 2C).

Furthermore, heterozygous sites in the 45S nrDNA sequences of the six *Allium* samples were investigated. Among the variations in the 45S nrDNA sequences, 21 heterozygous sites were observed in the 45S nrDNA sequences of farm collections JB and SA, containing two nucleotides at the same site. Each nucleotide of the two nucleotides in farm collections JB and SA was consistent with the nrDNA sequences of *AU* and *AM*, respectively, and depth ratio of each nucleotide type was about 7:3 in Farm-JB and 6:4 in Farm-SA overall (Fig 2B, Table 2).

**Table 2:** Variables and corresponding read mapping depths in 45S nrDNA sequences across the six *Allium* samples.

Nucleotide position Sample name	730	1837	1864	1876	1923	1936	2004	2009	2032	2172	2337	2424	2426	2690	2702	3009	3915	4443	4487	5196	5718
*AU*	A	A	A	T	G	-	A	A	T	C	C/T	T	T	C	A	A	A	C	C	G	C
*AM*	G	G	G	C	A	A	G	T	A	T	T	A	G	A	G	G	G	T	T	T	G/C
*AO*	G	G	G	C	A	A	G	T	A	T	T	A	G	A	G	G	G	T/C	T	T/G	G/C
Farm-TB	A	A	A	T	G	-	A	A	T	C	C	T	T	C	A	A	A	C	C	G	C
Farm-JB (hetero %)	A (66%)	A (61%)	A (61%)	T (63%)	G (64%)	- (64%)	A (63%)	A (63%)	T (63%)	C (63%)	C (63%)	T (69%)	T (69%)	C (72%)	A (72%)	A (69%)	A (65%)	C (70%)	C (70%)	G (68%)	C (69%)
	G (34%)	G (39%)	G (39%)	C (37%)	A (36%)	A (36%)	G (37%)	T (37%)	A (37%)	T (37%)	T (37%)	A (31%)	G (31%)	A (28%)	G (28%)	G (31%)	G (35%)	T (30%)	T (30%)	T (32%)	G (31%)
Farm-SA (hetero %)	A (56%)	A (53%)	A (52%)	T (54%)	G (54%)	- (57%)	A (57%)	A (57%)	T (58%)	C (59%)	C (57%)	T (59%)	T (58%)	C (62%)	A (62%)	A (61%)	A (53%)	C (54%)	C (56%)	G (57%)	C (60%)
	G (44%)	G (47%)	G (48%)	C (46%)	A (46%)	A (43%)	G (43%)	T (43%)	A (42%)	T (41%)	T (43%)	A (41%)	G (42%)	A (38%)	G (38%)	G (39%)	G (47%)	T (46%)	T (44%)	T (43%)	G (40%)

### Phylogenetic relationships of *Allium* species

We downloaded 19 previously reported plastome sequences of 12 *Allium* species from the NCBI GenBank. These included sequences from *A*. *microdictyon*, *A*. *ochotense*, *A*. *ullengense*, *A*. *victorialis*, *A*. *listera*, *A*. *ovalifolium*, *A*. *nanodes*, *A*. *prattii*, *A*. *tricoccum*, *A*. *neriniflorum*, *A*. *karataviense* and *A*. *cepa* (KM088013). Using these 19 downloaded plastomes and the six newly assembled plastomes from this study, we conducted a phylogenetic analysis to elucidate the phylogenetic relationships of the subgenus *Anguinum* of the genus *Allium*. The plastome-based phylogeny revealed that *AU* and Farm-TB formed a clade with three previously reported *A*. *ullengense*, and the clade was sister to the clade of *A*. *listera* and one *A*. *victorialis* (NC_037240). The other two *A*. *victorialis* were clustered with *A*. *microdictyon* and *A*. *ochotense*. Within the clade of *A*. *microdictyon* and *A*. *ochotense*, two farm collections, JB and SA, formed a clade with *AM*, previously reported *A*. *microdictyon* (OP743936) and *A*. *ochotense* (OP743938), and then the clade was sister to the clade of two A. ochotense (OP743947 and OP743948) (S2 Fig).

For the nrDNA-based analysis, we utilized the ITS1-5.8S-ITS2 sequences of 45S nrDNA, including the six *Allium* samples and 64 accessions from eight closely related *Allium* species (*A*. *microdictyon*, *A*. *ochotense*, *A*. *neriniflorum*, *A*. *ovalifolium*, *A*. *prattii*, *A*. *tricoccum*, *A*. *victorialis* and *A*. *listera*) obtained from NCBI. The ITS1-5.8S-ITS2 sequence of *A*. *cepa* (KM117265) was included as the outgroup. The *AM* and *AO* were located in the same clade with other sequences from *A*. *microdictyon*, *A*. *ochotense*, *A*. *listera* and *A*. *victorialis*. This clade was observed to be a sister group to the *A*. *tricoccum* clade. The *AU* and Farm-TB along with farm collections JB and SA, and two previously reported *A*. *ulleungense* accessions, formed a distinct clade (S2 Fig). A comparison of the plastome and nrDNA phylogenies showed that *A*. *ulleungense* was distinctly separated from other *Allium* species in the subgenus *Anguinum*. In addition, while the two farm collections, JB and SA, aligned closely with *AM* in the plastome phylogeny, they were closer to *AU* and Farm-TB in the nrDNA phylogeny (S2 Fig). As a result, among the three farm collections, Farm-TB was identified as *A*. *ulleungense*. However, Farm-JB and Farm-SA presented conflicting results between the plastome and nrDNA phylogenies (Figs 2C and S2). This result, combined with the results from heterozygous sites of 45S nrDNA in the two farm collections (Table 2), suggests that these two farm collections likely originated by hybridization between *A*. *ulleungense* as the paternal and *A*. *microdictyon* as the maternal species.

### Molecular marker design and validation

**Fig 3:**
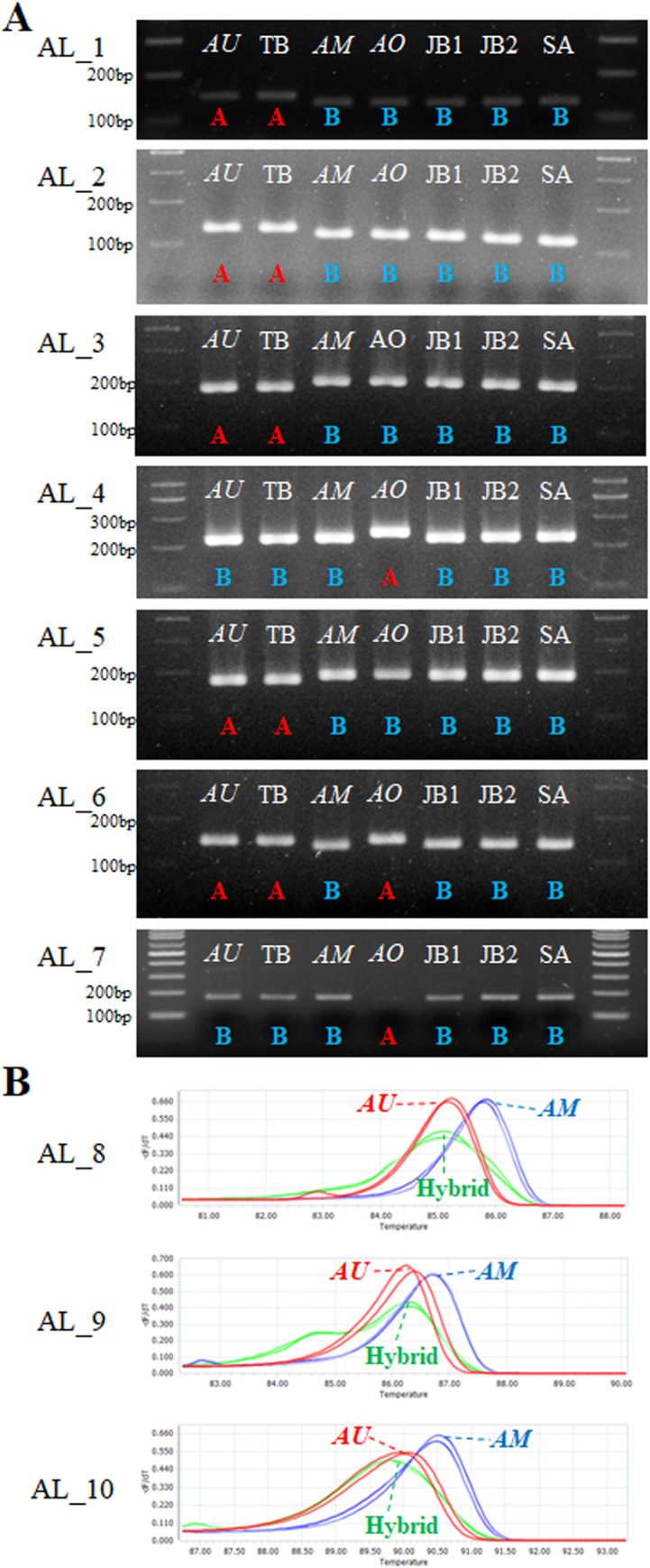
Validation results for molecular markers. (A) Gel electrophoresis result for PCR products derived from the molecular markers. (B) Melting peak results from HRM analysis using the HRM markers.

Based on sequence variations among six *Allium* accessions, ten molecular markers were designed to identify three *Allium* species: *AU*, *AM*, and *AO*. Six InDels located in the *rpoC2*, *rpoB–trnC*, *rps18–rpl20*, *trnV–rps12*, and *rps16–trnQ* regions, and one SNP located in the *psbC–trnS* region of the *Allium* plastomes were selected to design six codominant markers (AL_1, AL_2, AL_3, AL_4, AL_5, and AL_6) and one dominant marker (AL_7) (Table 3). Moreover, SNPs in the nrITS region of the 45S nrDNA, were used for the HRM analysis (Table 3). The ten designed markers were applied initially to verification with the six *Allium* samples. As the results, three *Allium* species, *AU*, *AM*, and *AO* could be identified using the seven designed markers from the plastome data (Fig 3A); *AO* (including Farm-TB) and *AM* could be identified using AL_1, AL_2, AL_3, AL_5, and AL_6, while *AO* could be distinguished using AL_4, AL_6, and AL_7 (Fig 3A). Furthermore, the two farm collections, JB and SA, which contained heterozygous sites in the nrDNA sequences, were identified using the melting peaks from three HRM analyses (Fig 3B). The melting peaks of the six *Allium* samples were categorized into three types: 1) *AU* and Farm-TB are denoted by red-colored peaks (*AU*), 2) *AM* and *AO* are denoted by blue-colored peaks (*AM*), and 3) the farm collections, JB and SA, are represented by green-colored peaks (Hybrid) (Fig 3B).

**Table 3:** Detailed information on the markers developed for *Allium* species.

Primer ID	Target genomes and genes		Marker type	Classification Product Size (bp)		Primer Sequence	
AL-1	Chloroplast	*rpoC2*	Codominant (InDel)	A: *AU*, Farm-TB	150	F:	AATGGGTGTGATTCGTTGGAC
				B: *AM*, *AO*, Farms-JB, SA	135	R:	ATTCATTTTCCCGGAGAGACAG
AL_2	Chloroplast	*rpoC2*	Codominant (InDel)	A: *AU*, Farm-TB	142	F:	TACGGGCCGAACCAAAACA
				B: *AM*, *AO*, Farms-JB, SA	127	R:	CGGCATTTTAATACCACCGGGA
AL_3	Chloroplast	*rpoB—trnC*	Codominant (InDel)	A: *AU*, Farm-TB	196	F:	CCGACGGGATACCGAATGAT
				B: *AM*, *AO*, Farms-JB, SA	213	R:	AGTTACGCAATTCAAGCGTATTCT
AL_4	Chloroplast	*rps18 –rpl20*	Codominant (InDel)	A: *AU*, *AM*, Farms-TB, JB, SA	233–236	F:	GCTGGTCCTAGAACCCGAAA
				B: *AO*	259	R:	GGAGAATGGACTCCGGGAAG
AL_5	Chloroplast	*trnV*–*rps12*	Codominant (InDel)	A: *AU*, Farm-TB	190	F:	GGGCAAAGGGATTGATCGAGAA
				B: *AM*, *AO*, Farms-JB, SA	200	R:	CCTTCTTCCACTCTGCCCC
AL_6	Chloroplast	*rps16 –trnQ-UUG*	Codominant (InDel)	A: *AU*, *AO*, Farm-TB	155	F:	TCTTTAGTCTCCATGTACAGTGTGT
				B: *AM*, Farms-JB, SA	143	R:	GTTCCATGAAATGTGAATTCTCGC
AL_7	Chloroplast	*psbC–trnS-UGA*	Dominant (SNP)	A: *AU*, *AM*, Farms-TB, JB, SA	184	F:	GACCCCACTTAGCTGAGATTTTTAAC
				B: *AO*	-	R:	GGTTCGAATCCCTCTCTCTCCT
AL_8	45S nrDNA	*ITS1-5*.*8s-ITS2*	HRM (SNP)	A: *AU*, Farm-TB B: *AM*, *AO*	A, A, T G, G, C	F:	GTTCGCCGCTTGTGACG
				C: Unknown D: Farms-JB, SA	A/G, A/G, T/C (hetero)	R:	GGAACCCATCCGAAAGGTCA
AL_9	45S nrDNA	*ITS1-5*.*8s-ITS2*	HRM (SNP)	A: *AU*, Farm-TB B: *AM*, *AO*	A, A, T G, T, A	F:	TGAAGCAAGAAGGAGAGGGG
				C: Unknown D: Farms-JB, SA	A/G, A/T, T/A (hetero)	R:	TGTCGCATTTCGCTACGTTC
AL_10	45S nrDNA	*ITS1-5*.*8s-ITS2*	HRM (SNP)	A: *AU*, Farm-TB B: *AM*, *AO*	A G	F:	CTTGGCTCGGTGTGTGGATT
				C: Farms-JB, SA	A/G (hetero)	R:	CCAAAGCTAAGAGCGCACAA

The ten developed markers were validated with extensive 49 samples of *Allium* species (S3 Table, S3 Fig). By Combining the results of these ten markers, the 49 samples were classified into five genotypes: *AU*-type, *AM*-type, *AO*-type, and two hybrid-estimated types (Tables 4 and S3). The two hybrid-estimated types were determined by their plastome type, and exhibited distinct melting peaks in the HRM analyses of AL_8 and AL_9 (S3 Table, S3 Fig). Among the 49 samples, fourteen samples, including *AU* and Farm-TB, were identified as *AU*-type, 22 individuals, including *AM*, were identified as *AM*-type; and only *AO* was categorized as *AO*-type (Tables 4 and S3, S3 Fig). The remaining 12 samples were identified as two hybrid-estimated types. Specifically, three samples (HJ1, HJ12, and HJ13) were identified as hybrids with the *AU*-type plastome (*AM* x *AU* hybrid), while nine samples (HC_OD1, HC_OD2, HC_OD3, HC_OD4, HC_OD5, JBds1, IJga, Farm-JB, and Farm-SA) were classified as hybrids with the *AM*-type plastome (*AU* x *AM* hybrid). The two farm collections, JB and SA, which had heterozygous sites in the nrDNA, were included in the *AU* x *AM* hybrid type (Tables 4 and S3, S3 Fig).

**Table 4:** Classification of 49 individuals of *Allium* species based on the genotypes from the developed molecular markers.

Represenatative accessions	Stem Color	Genotype for each marker										No. of collections
		AL_1	AL_2	AL_3	AL_4	AL_5	AL_6	AL_7	AL_8	AL_9	AL_10	
*AU*	G	A	A	A	A	A	A	A	A	A	A	14
*AM*	P	B	B	B	A	B	B	A	B	B	B	22
Hybrid (*AU* x *AM*)	G	A	A	A	A	A	A	A	H_1_	H_1_	A	3
Hybrid (*AM* x *AU*)	P	B	B	B	A	B	B	A	H_2_	H_2_	H	8
	G	B	B	B	A	B	B	A	H_2_	H_2_	H	1
*AO*	P	B	B	B	B	B	A	B	B	B	B	1

G: green, P: purple, H: hetero

## Discussion

### Genetic diversity of plastome and nrDNA among three *Allium* species

The *AU* (*A*. *ulleungense*) was previously recognized as AO (*A*. *ochotense*). However, Herdan et al. [5] and Prokhanov [20] argued that *AO* should be treated as two independent species based on their ploidy levels; diploid *AO* (= *A*. *latissimum*) and tetraploid *AO* [5,20]. Recently, the diploid *AO* was treated as a new species, endemic to Ulleung Island in South Korea [7]. According to Choi et al. [7], the *AU* (*A*. *ulleungense*) was clearly distinguished from *AM* (*A*. *microdictyon*) and *AO* (*A*. *ochotense*) by its morphological characters, such as relatively broader leaves and larger whitish perianth, chromosome number, and molecular evidence from nrITS and plastome data [7,21]. In this study, significant genetic diversity among *AU*, *AM* and *AO* was discovered through sequence variations and phylogenetic results based on the plastome and 45S nrDNA sequences. In plastomes of the three *Allium* species, about 180 SNPs and 80 InDels were found between *AU* and the other two species (*AM* and *AO*), and nearly 60 SNPs and 40 InDels were found between *AM* and *AO*, and the relationships among the three species were supported by 100% branch supports in the plastome phylogeny (Fig 2C). These results imply that the three *Allium* species are genetically differentiated from each other, and the taxonomic treatment of the *AU* is genetically supported.

However, two farm collections, JB and SA, showed conflicting results between plastome and 45S nrDNA data in sequence comparisons and phylogenetic analyses. The two farm collections were closer to *AM* in plastome data (Fig 2A and 2C), whereas they were identical with *AU* and Farm-TB in nrDNA data (Fig 2B and 2C). The discrepancy between plastome and nrDNA data is likely caused by a hybridization between *AU* and *AM*. While the plastome is maternally inherited in most angiosperms, 45S nrDNA is inherited from both parents [22,23]. The 45S nrDNA sequences have been used to study hybrids in various plants [24–26]. In this study, nucleotide type and read mapping depth of the 45S nrDNAs were analyzed to verify the possibility of crossing. The results indicated that two farm collections, JB and SA, displayed heterozygous sites containing both *AU*- and *AM*-types (Table 2). According to the heterozygous sites in the nrDNA sequences and both phylogenies based on plastome and nrDNA sequences, the farm collections JB and SA are likely to have originated from the hybridization between *AU* (paternally) and *AM* (maternally), but they may not be the F1 hybrids, having an uneven genetic proportion inherited from the two parental species (Table 2).

### Phylogenetic relationships of three *Allium* species

In a previous study of the subgenus *Anguinum* using sequences of the nrITS region and three plastid markers (*rps16* intron, *rbcL-atpB*, and *rpl32-trnL*), the phylogenetic relationships of three *Allium* species, *AM*, *AO*, and *A*. *victorialis*, were revealed [5]. In this study, we performed phylogenetic analyses using complete plastome and 45S nrDNA sequences of six *Allium* samples with other related species and showed that the *AU*, including *AU*, Farm-TB, and three previously reported *AU*, were clearly distinguished from *AM* and *AO*. These samples were sister to the clade of one *A*. *victorialis* and *A*. *listera* in the plastome phylogeny (S2 Fig), which was consistent with the previously constructed plastome phylogeny [21]. The sister relationship between AU and the clade of *A*. *victorialis*, *A*. *listera*, *AM*, and *AO* was supported in the nrITS tree (S2 Fig).

Based on a few DNA barcoding regions, the divergence time of the *AU* from other *Allium* species was previously estimated at approximately 2.54 million years ago (Ma) [5]. However, a recent study using plastome data indicated that the split between AU and other species, such as *A*. *listera*, *A*. *victorialis*, *AM*, and *AO*, occurred around 1.6 Ma [21]. The estimated time was almost consistent with the formation of Ulleung Island (1.5 Ma) [27,28]. We speculate that the genetic differentiation between *AU* and other *Allium* species shown in the phylogenetic analyses may be related to the geographical factor of Ulleung Island, where *AU* is distributed. Ulleung Island in South Korea is a volcanic island located far from the Korean peninsula [27,28], and several species originating from anagenetic speciation have been reported [29–31]. The conflicting positions of *AU* observed between the plastome and nrDNA phylogenies were similar to those observed in *Phedimus takesimensis* (Crassulaceae), where the origin of anagenetic speciation was revealed through a previous study [30]. Therefore, we speculate that *AU* may have undergone anagenetic speciation in the isolated environment of Ulleung Island like other endemic species on the island [29–31].

### Species identification and marker-assisted breeding of *Allium* species using molecular markers

Species identification in plants has predominantly relied on morphological characters, due to their highly polymorphic and easily observable nature. However, this traditional approach to species identification comes with limitations [32]. One major challenge is the presence of variation in plant morphology within the same species, which can lead to discrepancies in classification and create difficulties in precisely differentiating between closely related species [32]. To address these shortcomings and enhance the accuracy of species identification, a combination of molecular markers and morphological characters has become imperative [32]. In this study, we sought to overcome these challenges by applying molecular markers for species identification of *Allium* species. We developed ten molecular markers based on sequence variations in the plastome and nrDNA sequences of six *Allium* samples, providing a robust toolset to identify and distinguish three *Allium* species: *AU*, *AM*, and *AO* (Table 3). Moreover, three HRM markers developed in this study demonstrated their capability to detect hetero-genotypes of hybrid-estimated species (Table 3, Figs 3 and S3).

In the contemporary context of plant breeding, marker-assisted breeding (MAB) has emerged as a cutting-edge methodology with considerable advantages over conventional breeding approaches [33]. The utilization of molecular markers streamlines the breeding process, making it less time-consuming and reducing the influence of environmental factors on breeding outcomes [33]. Notably, MAB also permits the effective selection of recessive alleles and traits that are controlled by multiple genes, further enhancing breeding efficiency and precision [33]. Given the advantages of MAB and the performance of the newly developed molecular markers in this study, we believe that these markers will be instrumental for species identification and genotype assessment in *Allium* species.

## Conclusions

In this study, we investigated the genetic diversity among three *Allium* species by analyzing data from the plastome and 45S nrDNA. Our findings revealed substantial genetic variation among the species, leading to the classification of six individuals into three distinct types: *AU* (*A*. *ulleungense*), *AM* (*A*. *microdictyon*), and *AO* (*A*. *ochotense*). This observation underscores the significant genetic differentiation present among three *Allium* species. Consequently, we developed ten molecular markers based on sequence variations to facilitate the identification of these three species. To validate the efficacy of the developed markers, we examined a total of 49 individuals from the three species, categorizing them into five types: *AU*, *AM*, *AO*, and two hybrid-estimated types. The newly developed markers are expected to be valuable tools for both species identification and marker-assisted breeding of these species in the future.

## Supporting information

S1 Raw images (PDF)

S1 Table Information of generated NGS data from six *Allium* samples. (DOCX)

S2 Table Detail information of plastomes of six *Allium* samples. (DOCX)

S3 Table Genotyping results using ten molecular markers on 49 individuals of *Allium* species. (DOCX)

S1 Fig *Allium* samples collected from various locations. (A-F) Leaf samples of UL, TB, JB, SA, OD and JP respectively. (G) Green stem color of UL. (H) Purple stem color of OD.
(TIF)

S2 Fig Phylogenetic analyses of *Allium* species including other related *Allium* species based on plastome and 45S nrDNA sequences. Bootstrap values are indicated near the branches (indicated only if the bootstrap value is greater than 50). (A) Phylogenetic tree based on whole plastome sequences. (B) Phylogenetic tree based on ITS1-5.8S-ITS2 region of 45S nrDNA sequences.
(TIF)

S3 Fig Application of ten molecular markers on 49 individuals of *Allium* species. (A) Gel electrophoresis result of PCR product derived from molecular markers. (B) Melting peak results of HRM analysis using HRM markers.
(TIF)
