# Correction: Authentication of *Allium ulleungense*, *A*. *microdictyon* and *A*. *ochotense* based on super-barcoding of plastid genome and 45S nrDNA

**DOI:** 10.1371/journal.pone.0316742

**Published:** 2024-12-27

**Authors:** Minyoung Lee, Hyo Young Lee, Jong-Soo Kang, Hyeji Lee, Ki-Jin Park, Jee Young Park, Tae-Jin Yang

There is an error in affiliation 1 for authors Minyoung Lee, Jong-Soo Kang, Hyeji Lee, Jee Young Park and Tae-Jin Yang. The correct affiliation 1 is: Department of Agriculture, Forestry and Bioresources, Plant Genomics & Breeding Institute, Research Institute of Agriculture and Life Science, College of Agriculture & Life Sciences, Seoul National University, Seoul, Republic of Korea.
