# Supplementary figures and images for: Authentication of Allium ulleungense, A. microdictyon and A. ochotense based on super-barcoding of plastid genome and 45S nrDNA
Source: PLoS One. 2023 Nov 20;18(11):e0294457. doi: 10.1371/journal.pone.0294457 (PMC10659177; doi:10.1371/journal.pone.0294457)

Original uncropped gel images used in Fig 3

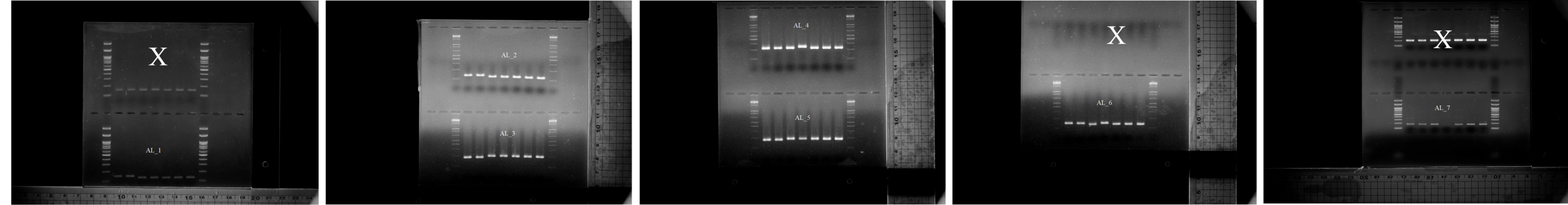

Original uncropped gel images used in S3 Fig

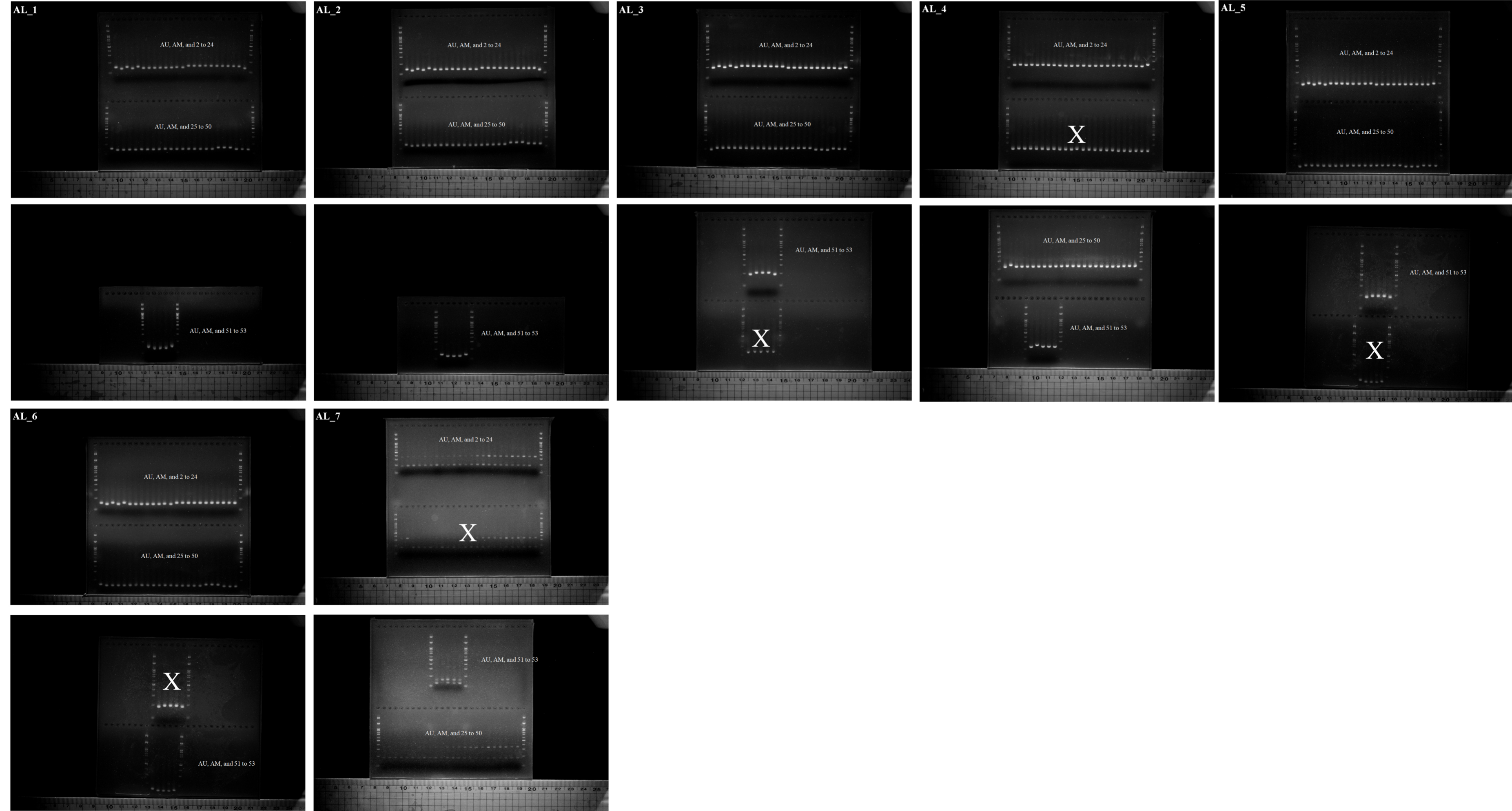

Supplement: S1 Raw images — (PDF) [file pone.0294457.s001.pdf]

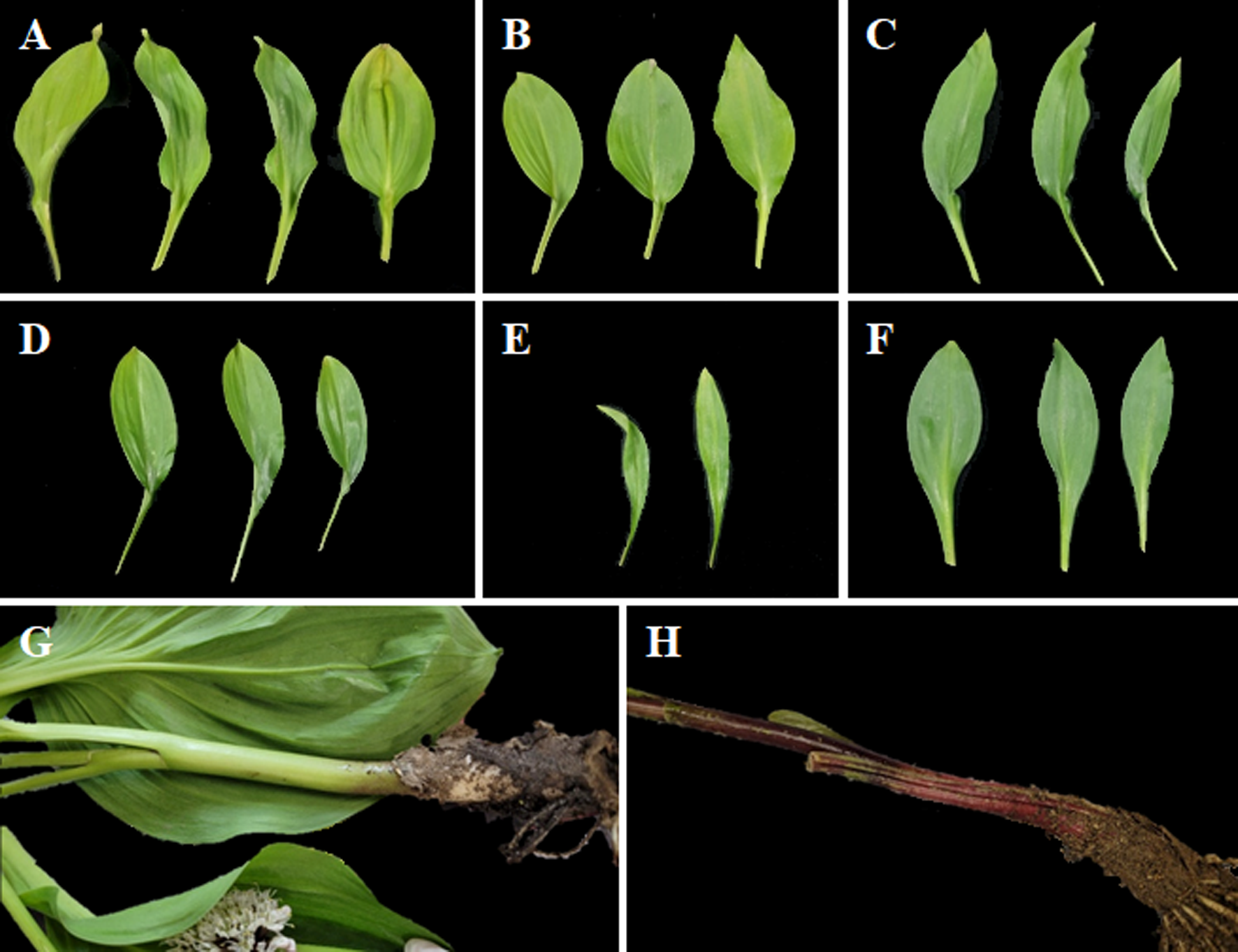

Supplement: S1 Fig — (A-F) Leaf samples of UL, TB, JB, SA, OD and JP respectively. (G) Green stem color of UL. (H) Purple stem color of OD. (TIF) [file pone.0294457.s005.tif]

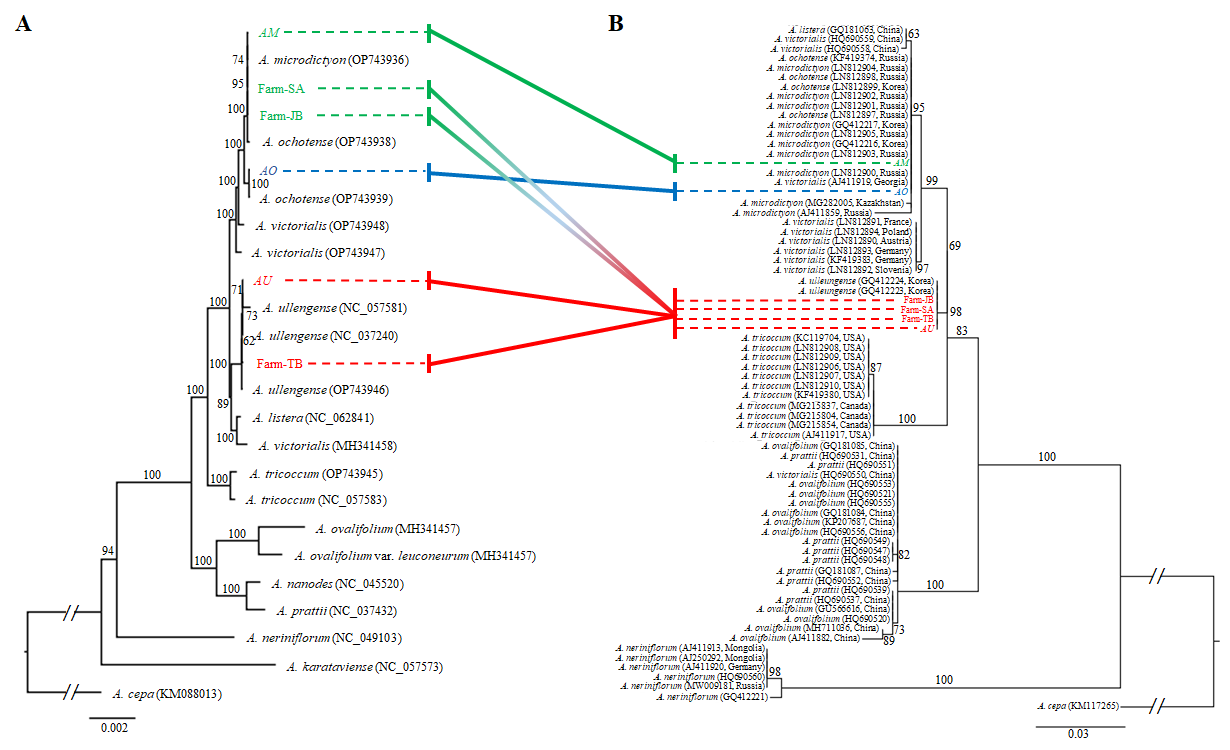

Supplement: S2 Fig — Bootstrap values are indicated near the branches (indicated only if the bootstrap value is greater than 50). (A) Phylogenetic tree based on whole plastome sequences. (B) Phylogenetic tree based on ITS1-5.8S-ITS2 region of 45S nrDNA sequences. (TIF) [file pone.0294457.s006.tif]

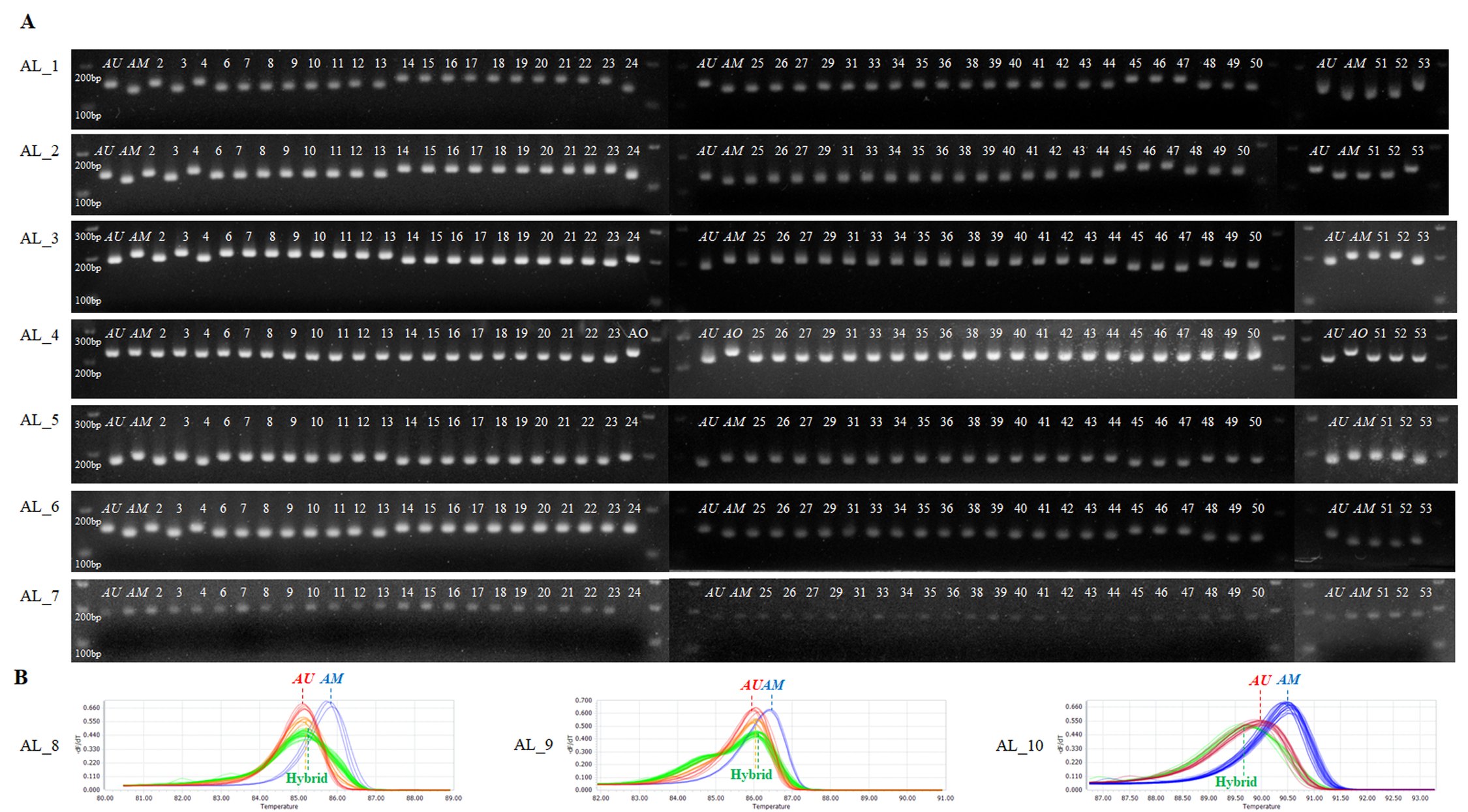

Supplement: S3 Fig — (A) Gel electrophoresis result of PCR product derived from molecular markers. (B) Melting peak results of HRM analysis using HRM markers. (TIF) [file pone.0294457.s007.tif]
