# Supplementary material for: Authentication of Allium ulleungense, A. microdictyon and A. ochotense based on super-barcoding of plastid genome and 45S nrDNA
Source: PLoS One. 2023 Nov 20;18(11):e0294457. doi: 10.1371/journal.pone.0294457 (PMC10659177; doi:10.1371/journal.pone.0294457)
Supplement: S1 Table — (DOCX) [file pone.0294457.s002.docx]

| **S1 Table.** Information of generated NGS data from six *Allium* samples. | | | |  | |  | |  | |  |
| --- | --- | --- | --- | --- | --- | --- | --- | --- | --- | --- |
| No. | Sample name | Raw reads # | Raw base (bp) | Trimmed reads # | | | Trimmed bases (bp) | | | |
| 1 | *AU* | 3,545,244 | 1,067,118,444 | 3,176,695 | 89.60% | | 840,747,774 | | 78.79% | |
| 2 | *AM* | 6,586,856 | 1,982,643,656 | 6,009,579 | 91.24% | | 1,624,900,238 | | 81.96% | |
| 3 | *AO* | 3,454,472 | 1,039,796,072 | 3,085,858 | 89.33% | | 802,862,924 | | 77.21% | |
| 4 | Farm-TB | 3,784,056 | 1,139,000,856 | 3,378,237 | 89.28% | | 891,393,349 | | 78.26% | |
| 5 | Farm-JB | 3,567,540 | 1,073,829,540 | 3,207,308 | 89.90% | | 838,078,013 | | 78.05% | |
| 6 | Farm-SA | 3,465,810 | 1,043,208,810 | 3,109,151 | 89.71% | | 810,272,851 | | 77.67% | |
