# Supplementary material for: Authentication of Allium ulleungense, A. microdictyon and A. ochotense based on super-barcoding of plastid genome and 45S nrDNA
Source: PLoS One. 2023 Nov 20;18(11):e0294457. doi: 10.1371/journal.pone.0294457 (PMC10659177; doi:10.1371/journal.pone.0294457)
Supplement: S2 Table — (DOCX) [file pone.0294457.s003.docx]

| **S2 Table.** Detail information of plastomes of six *Allium* samples. | | | | | | | |
| --- | --- | --- | --- | --- | --- | --- | --- |
| Sample name | Length (bp) | LSC (bp) | IR (bp) | SSC (bp) | Protein-coding gene | tRNA | rRNA |
| *AU* | 154,047 | 83,142 | 26,526 | 17,853 | 86 | 38 | 8 |
| *AM* | 153,562 | 82,622 | 26,542 | 17,856 | 86 | 38 | 8 |
| *AO* | 153,125 | 82,183 | 26,542 | 17,858 | 86 | 38 | 8 |
| Farm-TB | 154,051 | 83,147 | 26,526 | 17,852 | 86 | 38 | 8 |
| Farm-JB | 153,560 | 82,620 | 26,542 | 17,856 | 86 | 38 | 8 |
| Farm-SA | 153,561 | 82,621 | 26,542 | 17,856 | 86 | 38 | 8 |
