# Supplementary material for: Authentication of Allium ulleungense, A. microdictyon and A. ochotense based on super-barcoding of plastid genome and 45S nrDNA
Source: PLoS One. 2023 Nov 20;18(11):e0294457. doi: 10.1371/journal.pone.0294457 (PMC10659177; doi:10.1371/journal.pone.0294457)
Supplement: S3 Table — (DOCX) [file pone.0294457.s004.docx]

| **S3 Table.** Genotyping results using ten molecular markers on 49 individuals of *Allium* species. | | | | | | |  |  |  |  |  |  |  |
| --- | --- | --- | --- | --- | --- | --- | --- | --- | --- | --- | --- | --- | --- |
| Sample No. | Sample ID | Collection site | Pheno-  type (stem color) | Genotyping results | | | | | | | | | |
|  |  |  |  | AL_1 | AL_2 | AL_3 | AL_5 | AL_6 | AL_4 | AL_7 | AL_8 | AL_9 | AL_10 |
| 1 | ***AU*** | Ulleung-gun, Gyeongsangbuk-do | G | A | A | A | A | A | A | A | A | A | A |
| 2 | **Farm-TB** | Taebaek-si, Gangwon-do | G | A | A | A | A | A | A | A | A | A | A |
| 3 | JBgp1 | Jinbu-myeon, Pyeongchang-gun, Gangwon-do | P | B | B | B | B | B | A | A | B | B | B |
| 4 | HJ1 | Bongpyeong-myeon, Pyeongchang-gun, Gangwon-do | G | A | A | A | A | A | A | A | C | C | A |
| 5 | ***AM*** | Bongpyeong-myeon, Pyeongchang-gun, Gangwon-do | P | B | B | B | B | B | A | A | B | B | B |
| 6 | HJ2 | Bongpyeong-myeon, Pyeongchang-gun, Gangwon-do | P | B | B | B | B | B | A | A | B | B | B |
| 7 | HJ3 | Bongpyeong-myeon, Pyeongchang-gun, Gangwon-do | P | B | B | B | B | B | A | A | B | B | B |
| 8 | HJ4 | Bongpyeong-myeon, Pyeongchang-gun, Gangwon-do | P | B | B | B | B | B | A | A | B | B | B |
| 9 | HJ5 | Bongpyeong-myeon, Pyeongchang-gun, Gangwon-do | P | B | B | B | B | B | A | A | B | B | B |
| 10 | HJ6 | Bongpyeong-myeon, Pyeongchang-gun, Gangwon-do | P | B | B | B | B | B | A | A | B | B | B |
| 11 | HJ7 | Bongpyeong-myeon, Pyeongchang-gun, Gangwon-do | P | B | B | B | B | B | A | A | B | B | B |
| 12 | HJ8 | Bongpyeong-myeon, Pyeongchang-gun, Gangwon-do | P | B | B | B | B | B | A | A | B | B | B |
| 13 | HJ9 | Bongpyeong-myeon, Pyeongchang-gun, Gangwon-do | P | B | B | B | B | B | A | A | B | B | B |
| 14 | HJ10 | Bongpyeong-myeon, Pyeongchang-gun, Gangwon-do | G | A | A | A | A | A | A | A | A | A | A |
| 15 | HJ11 | Bongpyeong-myeon, Pyeongchang-gun, Gangwon-do | G | A | A | A | A | A | A | A | A | A | A |
| 16 | HJ12 | Bongpyeong-myeon, Pyeongchang-gun, Gangwon-do | G | A | A | A | A | A | A | A | C | C | A |
| 17 | HJ13 | Bongpyeong-myeon, Pyeongchang-gun, Gangwon-do | G | A | A | A | A | A | A | A | C | C | A |
| 18 | HJ14 | Bongpyeong-myeon, Pyeongchang-gun, Gangwon-do | G | A | A | A | A | A | A | A | A | A | A |
| 19 | HJ15 | Bongpyeong-myeon, Pyeongchang-gun, Gangwon-do | G | A | A | A | A | A | A | A | A | A | A |
| 20 | HJ16 | Bongpyeong-myeon, Pyeongchang-gun, Gangwon-do | G | A | A | A | A | A | A | A | A | A | A |
| 21 | TB2 | Taebaek-si, Gangwon-do | G | A | A | A | A | A | A | A | A | A | A |
| 22 | SC1 | Hajang-myeon, Samcheok-si, Gangwon-do | G | A | A | A | A | A | A | A | A | A | A |
| 23 | SC2 | Hajang-myeon, Samcheok-si, Gangwon-do | G | A | A | A | A | A | A | A | A | A | A |
| 24 | ***AO*** | Japan | P | B | B | B | B | A | B | B | B | B | B |
| 25 | **Farm-JB** | Jinbu-myeon, Pyeongchang-gun, Gangwon-do | P | B | B | B | B | B | A | A | D | D | C |
| 26 | JBds1 | Jinbu-myeon, Pyeongchang-gun, Gangwon-do | P | B | B | B | B | B | A | A | D | D | C |
| 27 | IJgd1 | Inje-eup, Inje-gun, Gangwon-do | P | B | B | B | B | B | A | A | B | B | B |
| 29 | IJgd2 | Inje-eup, Inje-gun, Gangwon-do | P | B | B | B | B | B | A | A | B | B | B |
| 31 | IJgd3 | Inje-eup, Inje-gun, Gangwon-do | P | B | B | B | B | B | A | A | B | B | B |
| 33 | IJgd4 | Inje-eup, Inje-gun, Gangwon-do | P | B | B | B | B | B | A | A | B | B | B |
| 34 | IJga | Inje-eup, Inje-gun, Gangwon-do | P | B | B | B | B | B | A | A | D | D | C |
| 35 | **Farm-SA** | Buk-myeon, Inje-gun, Gangwon-do | P | B | B | B | B | B | A | A | D | D | C |
| 36 | Ijyd | Buk-myeon, Inje-gun, Gangwon-do | P | B | B | B | B | B | A | A | B | B | B |
| 38 | IJgr | Girin-myeon, Inje-gun, Gangwon-do | P | B | B | B | B | B | A | A | B | B | B |
| 39 | JBgp2 | Jinbu-myeon, Pyeongchang-gun, Gangwon-do | P | B | B | B | B | B | A | A | B | B | B |
| 40 | JBgp3 | Jinbu-myeon, Pyeongchang-gun, Gangwon-do | P | B | B | B | B | B | A | A | B | B | B |
| 41 | JBgp4 | Jinbu-myeon, Pyeongchang-gun, Gangwon-do | P | B | B | B | B | B | A | A | B | B | B |
| 42 | JBgp5 | Jinbu-myeon, Pyeongchang-gun, Gangwon-do | P | B | B | B | B | B | A | A | B | B | B |
| 43 | JBgp6 | Jinbu-myeon, Pyeongchang-gun, Gangwon-do | P | B | B | B | B | B | A | A | B | B | B |
| 44 | JBgp7 | Jinbu-myeon, Pyeongchang-gun, Gangwon-do | P | B | B | B | B | B | A | A | B | B | B |
| 45 | HC_UL1 | Nae-myeon, Hongcheon-gun, Gangwon-do | G | A | A | A | A | A | A | A | A | A | A |
| 46 | HC_UL2 | Nae-myeon, Hongcheon-gun, Gangwon-do | G | A | A | A | A | A | A | A | A | A | A |
| 47 | HC_UL3 | Nae-myeon, Hongcheon-gun, Gangwon-do | G | A | A | A | A | A | A | A | A | A | A |
| 48 | HC_OD1 | Nae-myeon, Hongcheon-gun, Gangwon-do | G | B | B | B | B | B | A | A | D | D | C |
| 49 | HC_OD2 | Nae-myeon, Hongcheon-gun, Gangwon-do | P | B | B | B | B | B | A | A | D | D | C |
| 50 | HC_OD3 | Nae-myeon, Hongcheon-gun, Gangwon-do | P | B | B | B | B | B | A | A | D | D | C |
| 51 | HC_OD4 | Nae-myeon, Hongcheon-gun, Gangwon-do | P | B | B | B | B | B | A | A | D | D | C |
| 52 | HC_OD5 | Nae-myeon, Hongcheon-gun, Gangwon-do | P | B | B | B | B | B | A | A | D | D | C |
| 53 | CC | Sabuk-myeon, Chuncheon-si, Gangwon-do | G | A | A | A | A | A | A | A | A | A | A |

Note: P and G indicate stem color of each sample which was purple or green. A and B indicate two haplotypes in the target plastome variations. A, B, C and D indicate 45S nrDNA SNP genotypes: A and B indicate *A. ulleungense* and *A. microdictyon* genotype, respectively. C and D indicate heterozygous genotype with *A. ulleungense*-plastid type and *A. microdictyon*-plastid type, respectively.
